# Targeting Triple NK Cell Suppression Mechanisms: A Comprehensive Review of Biomarkers in Pancreatic Cancer Therapy

**DOI:** 10.3390/ijms26020515

**Published:** 2025-01-09

**Authors:** Sara Fanijavadi, Mads Thomassen, Lars Henrik Jensen

**Affiliations:** 1 Cancer Polyclinic, Levanger Hospital, 7601 Levanger, Norway; 2 Department of Oncology, Vejle Hospital, University Hospital of Southern Denmark, 7100 Vejle, Denmark; lars.henrik.larsen@rsyd.dk; 3 Department of Clinical Genetics, Odense University Hospital, 5000 Odense, Denmark; mads.thomassen@rsyd.dk; 4 Department of Clinical Research, University of Southern Denmark, 5230 Odense, Denmark; 5 Department of Oncology, Institute of Regional Health Research, University of Southern Denmark, 7100 Vejle, Denmark

**Keywords:** pancreatic cancer, natural killer cell, tumor microenvironment, biomarker, therapeutic resistance, personalized oncology

## Abstract

Pancreatic ductal adenocarcinoma (PDAC) is an aggressive cancer with poor outcomes due to frequent recurrence, metastasis, and resistance to treatment. A major contributor to this resistance is the tumor’s ability to suppress natural killer (NK) cells, which are key players in the immune system’s fight against cancer. In PDAC, the tumor microenvironment (TME) creates conditions that impair NK cell function, including reduced proliferation, weakened cytotoxicity, and limited tumor infiltration. This review examines how interactions between tumor-derived factors, NK cells, and the TME contribute to tumor progression and treatment resistance. To address these challenges, we propose a new “Triple NK Cell Biomarker Approach”. This strategy focuses on identifying biomarkers from three critical areas: tumor characteristics, TME factors, and NK cell suppression mechanisms. This approach could guide personalized treatments to enhance NK cell activity. Additionally, we highlight the potential of combining NK cell-based therapies with conventional treatments and repurposed drugs to improve outcomes for PDAC patients. While progress has been made, more research is needed to better understand NK cell dysfunction and develop effective therapies to overcome these barriers.

## 1. Introduction

Pancreatic ductal adenocarcinoma (PDAC) is the most prevalent and one of the deadliest forms of pancreatic cancer, with a poor prognosis due to factors such as late-stage diagnosis, high recurrence rates, metastasis, and intratumoral heterogeneity [1]. The aggressiveness of PDAC is further compounded by cancer cell plasticity, including epithelial-to-mesenchymal transition (EMT), a process by which cells acquire mesenchymal properties associated with increased invasiveness and resistance [2]. Additionally, pancreatic stellate cells (PSCs), cancer stem cells (CSCs), and the immunosuppressive, desmoplastic, and hypovascular tumor microenvironment (TME) contribute significantly to treatment challenges and chemoresistance [3]. PDAC typically develop mechanisms that suppress natural killer (NK) cell function. As a result, PDAC remains resistant to current therapies. NK cells, which are cytotoxic lymphocytes, play a crucial role in eliminating tumor cells and virally infected cells as part of the innate immune response. Several mechanisms in the TME are responsible for NK cell suppression. For example, cancer-associated fibroblasts (CAFs), activated by transforming growth factor beta (TGF-β) from stromal cells, create an immunosuppressive and fibrotic environment, reducing NK cell receptor expression (e.g., NK group 2D receptor (NKG2D)) and cytotoxic factors like interferon gamma (IFN-γ), perforin, and granzyme B. This fibrotic environment also contributes to diminished NK cell tumor infiltration and chemotherapy resistance [4,5,6,7].

Current standard therapies for metastatic PDAC, such as a combination of 5-fluorouracil, leucovorin, irinotecan, and oxaliplatin (FOLFIRINOX) and gemcitabine/nano albumin- bound paclitaxel, provide only limited survival benefits compared to gemcitabine monotherapy (11.1 vs. 6.8 months for FOLFIRINOX vs. gemcitabine) [8]. While one study reported longer median progression-free survival (mPFS) (5.2 months vs. 2.9 months) with prior FOLFIRINOX, the median overall survival (mOS) did not show a significant difference compared to gemcitabine/nab-paclitaxel (12.3 vs. 11.3 months) [8,9,10].

Chemoresistance in PDAC is driven by various mechanisms [11], many of which are linked to the suppression of NK cell activity, resulting in a diminished ability to target and eliminate tumor cells. Gene expression microarray analyses of PDAC cell lines have identified more than 165 genes associated with drug resistance. These genes are involved in diverse cellular processes, including antioxidant activity, apoptosis, cell cycle regulation, and signal transduction [12]. Additionally, microRNAs (miRNAs) play a critical role in modulating chemoresistance in PDAC. Certain miRNAs like miR-181c, highly expressed in advanced stages of the disease, contribute to increased resistance to chemotherapeutic agents such as gemcitabine, 5-FU, and paclitaxel. Conversely, miRNAs like miR-509-5p and miR-1243 inhibit the EMT. Overexpression of these miRNAs sensitizes PDAC cells to gemcitabine, highlighting their potential as therapeutic targets to overcome chemoresistance [13,14]. Notably, chemotherapy can stimulate the immune system by inducing immunogenic cell death (ICD), which releases damage-associated molecular patterns (DAMPs) such as calreticulin (CRT), adenosine triphosphate (ATP), and high mobility group box 1 (HMGB1). These DAMPs act as extrinsic factors, enhancing NK cell activation and their ability to target tumor cells [15,16].

Chemotherapy also influences NK cell activity by modulating their receptor expression, including activating receptors like NKG2D and NK cell p44-related proteins (NKp44), and inhibitory receptors such as killer cell immunoglobulin-like receptors (KIRs). These receptor changes, regulated by intrinsic factors within NK cells, are critical for their ability to recognize and eliminate tumor cells. The interplay between intrinsic factors, such as NK cell receptor signaling pathways and metabolic processes, and extrinsic factors from the TME, like DAMPs and secreted proteins, ultimately shapes NK cell phenotypes and their effectiveness in the antitumor immune response [3,17,18,19].

Cancer stem cells (CSCs) are also critical contributors to drug resistance in PDAC. These cells are a group of cancer cells with stem-like traits that drive tumor growth, therapy resistance, relapse, and metastasis. Interestingly, EMT transcription factors contribute to these stem-like properties and chemoresistance, but their role in PDAC metastasis is still debated [20]. These cells are capable of overexpressing ATP-binding cassette (ABC) transporters, enzymes like aldehyde dehydrogenases (involved in drug metabolism), and poly (ADP-ribose) polymerases (involved in DNA damage repair). Key ABC transporters in PDAC include breast cancer resistance protein (BCRP), P-glycoprotein (P-gp), and multidrug resistance protein (MRP), all of which are involved in drug transport and contribute to the chemoresistant phenotype [21]. Additionally, PDAC stemness not only fosters drug resistance but also influences the immune response, impairing NK cell infiltration into the tumor and contributing to a poor prognosis [22]. Furthermore, the two major molecular subtypes of PDAC—classical epithelial (E) and quasi-mesenchymal (QM)—as well as the key role of EMT, have been shown to impact treatment responses in both preclinical and clinical settings [23]. Studies have demonstrated that FOLFIRINOX combination chemotherapy promotes a shift toward a more QM phenotype, where cells undergo EMT. This shift is associated with increased chemoresistance and the acquisition of an EMT-like phenotype in cancer cells. For example, reverse transcription–polymerase chain reaction (RT-PCR) analysis revealed high levels of Zinc-finger E-box-binding homeobox (ZEB1) and vimentin in chemoresistant cells. Furthermore, silencing ZEB1 restored E-cadherin expression, leading to increased NK cell activity and drug sensitivity, highlighting the molecular link between EMT and chemoresistance [24,25].

**Figure 1:**
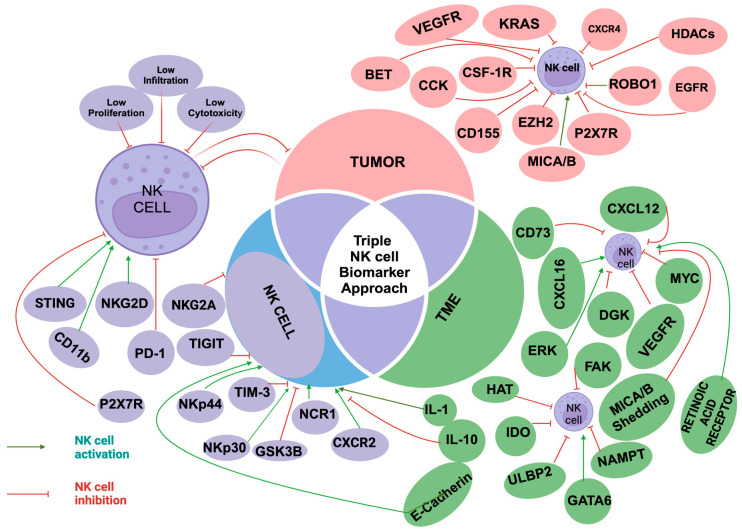
Triple NK cell biomarker approach.

This review will focus on the diagnostic and therapeutic potential of NK cells in overcoming PDAC resistance. We will examine three key components: NK cell phenotypes, tumor characteristics, and changes in the TME. Specifically, we will discuss mechanisms of NK cell suppression, including alterations in NK cell abundance, cytotoxicity, and tumor infiltration. By using a “triple NK cell biomarker approach (Figure 1) we aim to identify biomarkers (Table 1) and targets associated with NK cell cytotoxicity in tumor cells, the TME, and NK cells. This integrated approach has the potential to provide critical insights into overcoming PDAC chemoresistance and facilitating the development of personalized therapeutic strategies.

This figure illustrates the three key components: the tumor, the tumor microenvironment (TME), and their interaction with NK cell activity. It highlights the three main mechanisms of NK cell suppression—reduced NK cell proliferation, cytotoxicity, and infiltration. Additionally, the figure shows various tumor and TME elements and markers that can either activate or suppress NK cells. Green Ovals: Distinct biomarkers derived from the TME; Violet Ovals: Biomarkers present on NK cells; Pink Ovals: Biomarkers originating from tumor cells; Green Arrows signify that the biomarker can activate NK cells; Red Stop Arrows indicate that the biomarker can suppress NK cell activity.

**Ag:** Antigen; **BET:** Bromodomain and extra terminal; CAF: Cancer-associated fibroblast; **CCK**: Cholecystokinin; **CD**: Cluster of differentiation; CpG: Cytosine phosphate guanine; **CSF**: Colony-stimulating factor; **CXCL**: Chemokine (C-X-C motif) Ligand; **CXCR**: Chemokine (C-X-C motif) Receptor; **DGK**: Diacylglycerol kinase; **DNAM-1:** DNAX accessory molecule-1**; ECM:** Extracellular matrix; **EGFR**: Epidermal growth factor; **ERK**: Extracellular signal-regulated kinase; **EZH2**: Enhancer of zeste homolog 2; **FAK**: Focal adhesion kinase; **GATA6**: Gata binding protein 6; **GSK3B**: Glycogen synthase kinase three beta; **HAT**: Histone acetyltransferase; **HDACs**: Histone deacetylases; **IDO**: Indoleamine-2, 3-dioxygenase; **IFNγ:** Interferon gamma; **IL**: Interleukin; **KIR:** Killer cell immunoglobulin-like receptor; **KRAS**: Kirsten rat sarcoma virus; LAG-3: Lymphocyte-activation gene 3; **MICA/B**: Major histocompatibility complex class I chain-related protein A and B; **miRNAs**: MicroRNA; **MYC**: MYC proto-oncogene; **NAMPT**: Nicotinamide phosphoribosyltransferase; **NCR**: Natural cytotoxicity receptor; **NKA**: NK cell activity; **NKG2A**: NK group 2A receptor; **NKG2D**: NK group 2D receptor; **NKP30**: NK cell p30-related proteins; **NKP44**: NK cell p44-related proteins; **P2 × 7**: Purinergic receptor 7; **P53:** Tumor protein p53; **PD-1**: Programmed cell death protein 1; **PSCs:** Pancreatic stellate cells; **PTEN:** Phosphatase and tensin homolog; **ROBO1**: Roundabout Guidance Receptor 1; **Shh:** Sonic hedgehog; **Src**: Steroid receptor coactivator **STAT**: Signal transducers and activators of transcription; **STING**: Stimulator of interferon response gene; **TGF-β:** transforming growth factor beta; **TIGIT**: T-cell immunoreceptor with Ig and ITIM domains; **TIM-3:** T-cell immunoglobulin- and mucin domain-containing 3 receptor**; TME**: Tumor microenvironment; **ULBP2**: UL-16 binding protein-2; **VEGFR**: Vascular endothelial growth factor.

**Table 1:** NK cell applications in the management of treatment-resistant pancreatic ductal adenocarcinoma (PDAC), using triple biomarker approach.

Biomarker/Target	Source	Impact on NK Cells	Therapeutic Approach	References
BET	Tumor cell	Inhibitory	Bromodomain inhibitors (increasing NKGD ligand MICA expression).	[26]
CCK	Tumor cell	Inhibitory	CCK inhibitors	[27,28]
CD11b	NK cell	Activating	CD11b agonists	[29,30]
CD155	Tumor cell	Inhibitory	Targeting alternating splicing of CD155	[31]
CD73	TME	Inhibitory	CD73 inhibitors or Diclofenac	[32,33]
CSF-1R	Tumor cell	Inhibitory	CSF-1R inhibitors	[34,35,36]
CXCL12	TME	Inhibitory	CXCL12 inhibitors	[37,38]
CXCL16	TME	Activating	NRP-body	[39]
CXCR2	NK cell	Activating	IL-1 therapy	[40,41]
CXCR4 (ligand: CXCL12)	Tumor cell	Inhibitory	CXCR4 antagonists	[6,41]
DAP1	Tumor cell	Activating	Inducing the overexpression	[42]
DAP3	Tumor cells	Inhibitory	DAP3 inhibitors	[43]
DGK	TME	Inhibitory	DGK inhibitors	[44]
E-cadherin	TME	Activating	EZH2 inhibitors, E-cadherin upregulation	[45,46]
EGFR-mutation	Tumor cell	Inhibitory	Cetuximab + IL-21	[47]
ERK	TME	Activating	NRP-body	[39]
EZH2	Tumor cell	Inhibitory	EZH2 knockdown or inhibitors, microRNA-26a, EZH2 blockade trametinib/palbociclib	[46,48]
FAK	TME	Inhibitory	FAK inhibitors	[49,50]
GATA6	TME	Activating	GATA6 inhibitors	[51,52]
GSK3B	NK cell	Inhibitory	GSK3B inhibitors	[53]
HAT	TME	Inhibitory	HAT inhibitors, curcumin	[54]
HDACs	Tumor cell	Inhibitory	HDAC inhibitors	[53]
IDO	TME	Inhibitory	IDO inhibitors	[55,56]
IL-1	TME	Activating	IL-1 therapy	[57,58,59]
IL-10	TME	Inhibitory	IL-10 inhibitors	[60,61,62]
KRAS	Tumor cell	Inhibitory	NK adoptive cell transfer, NK cell infiltration inducers	[63,64]
MICA/B shedding	TME	Inhibitory	MICA/B inhibitors	[65]
MICA/B Expressen	Tumor cell	Activating	Low-dose gemcitabine	[50,66]
MYC	TME	Inhibitory	MYC inhibitors	[2,67]
NAMPT	TME	Inhibitory	NAMP inhibitors + metformin, STING agonists	[68,69]
NCR1(NKp46)	NK cell	Activating	IDO inhibitors, STAT3 inhibitors, NK cell engagers	[55,70]
NKG2A	NK cell	Inhibitory	NK cell engagers, NKG2A blockade	[70,71]
NKG2D	NK cell	Activating	NK cell engagers	[70,72]
NKp30	NK cell	Activating	NK cell engagers	[70]
NKp44(NCR2)	NK cell	Activating	NK cell engagers	[70]
P2X7R	Tumor cell NK cell	Inhibitory	P2X7 inhibitors	[73,74,75]
PD-1	NK cell	Inhibitory	PD-1 checkpoint inhibitors + IL-6 inhibitors or Lenvatinib; Lenvatinib + GVAX + CSF-1R inhibitors	[76]
Retinoic acid receptors	TME	Activating	ATRA	[77]
ROBO1	Tumor cell	Inhibitory	ROBO1-targeted CAR NK therapy or NK cell infusion	[59,78,79]
STING	TME	Activating	STING agonists + gemcitabine	[80,81]
TIGIT	NK cell	Inhibitory	Trastuzumab + rituximab, and anti-TIGIT monoclonal antibody	[82]
TIM-3	NK cell	Inhibitory	TIM-3 targeting	[83,84]
ULBP2	TME	Inhibitory	ULBP2 downregulation by gemcitabine	[85]
VEGFR	TME Tumor cell	Inhibitory	VEGFR inhibitors + anti-PD-1	[76]

This table outlines the design of a triple NK cell biomarker approach for biomarker studies, providing a comprehensive framework for assessing NK cell-related biomarkers from different sources (tumor, TME, or NK cells). The first column (“Biomarker/Target”) lists the biomarkers, while the second column (“Source”) indicates their origin (Tumor, TME or NK cells). These biomarkers are suggested for use in diagnosing NK cell status and as potential targets for a multitargeting therapeutic approach. The third column (“Impact on NK Cell Activity”) describes whether the biomarker exerts an inhibitory or activating effect on NK cells. An inhibitory effect means that the biomarker’s normal expression suppresses NK cell activity, while an activating effect indicates that the biomarker promotes NK cell function. The final column (“Therapeutic Approach”) describes potential interventions to counteract NK cell suppression. For example, targeting BET (a tumor cell biomarker) with bromodomain inhibitors can enhance MICA expression on tumor cells, thereby improving NK cell recognition and tumor cell killing. The table emphasizes the importance of the triple NK cell biomarker approach, which considers biomarkers from multiple sources rather than focusing on a single source. This comprehensive approach helps avoid misleading conclusions. For instance, the role of CD11b+ NK cells are context-dependent and shaped by environmental signals. While increased CD11b+ NK cells could signify immune activation, their presence in an immunosuppressive TME may reflect cytotoxic dysfunction. If CD11b is low, CD11b agonists could restore NK cell function, but if it is high, it indicates that CD11b is ineffective, and other biomarkers need to be identified and targeted for effective therapy.

**ATRA:** All trans retinoic acid; **BET:** Bromodomain and extra terminal; **CAR-NK therapy**: Chimeric antigen receptor natural killer cell therapy; **CCK**: Cholecystokinin; **CD**: Cluster of differentiation; **CSF**: Colony-stimulating factor; **CXCL**: Chemokine (C-X-C motif) ligand; **CXCR**: Chemokine (C-X-C motif) receptor ; **DAP**: Death-Associated Protein; **DGK**: Diacylglycerol kinase ; **EAAL**: Expanded activated allogeneic lymphocytes; **EGFR**: Epidermal growth factor; **ERK**: Extracellular signal-regulated kinase; **EZH2**: Enhancer of zeste homolog 2; **FAK**: focal adhesion kinase; **GATA6**: Gata binding protein 6; **GSK3B**: Glycogen synthase kinase three beta; **GVAX**: Granulocyte-macrophage colony-stimulating factor gene transduced autologous pancreatic cancer Vaccine; **HAT**: Histone acetyltransferase; **HDACs**: Histone deacetylases; **IDO**: Indoleamine-2, 3-dioxygenase; **IL**: Interleukin; **IRE:** Irreversible electroporation; **KRAS**: Kirsten rat sarcoma virus; **MICA/B**: Major histocompatibility complex class I chain-related protein A and B; **MYC**: MYC proto-oncogene; **NAMPT**: Nicotinamide phosphoribosyltransferase; **NCR**: Natural cytotoxicity receptor; **NKA**: NK cell activity; **NKG2A**: NK group 2A receptor; **NKG2D**: NK group 2D receptor; **NKP30**: NK cell p30-related proteins; **NKP46**: NK cell p46-related proteins; **NRP-body**: NK cell-recruiting protein-conjugated antibodies; **P2X7**: Purinergic receptor 7; **PD-1**: Programmed cell death protein 1; **PDAC**: Pancreatic ductal adenocarcinoma; **PKM2:** Pyruvate kinase muscle 2; **ROBO1**: Roundabout Guidance Receptor 1; **STAT**: Signal transducers and activators of transcription; **STING**: Stimulator of interferon response gene; **TIGIT**: T-cell immunoreceptor with Ig and ITIM domains; **TME**: Tumor microenvironment; **ULBP2**: UL-16 binding protein-2 ; **VEGFR**: Vascular endothelial growth factor.

## 2. NK Cells: Diagnostic Potential

**Figure 2:**
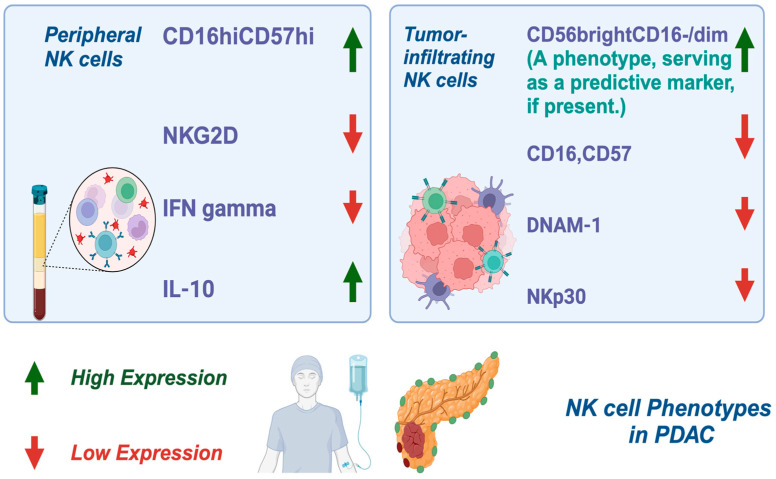
NK cell phenotypes in PDAC.

NK cells are critical lymphocytes in the innate immune system, recognized for their pivotal role in tumor immunosurveillance. They function by balancing of activating and inhibitory receptors, including cytokine receptors and those involved in antibody-dependent cell-mediated cytotoxicity (ADCC) [17,86]. In PDAC, peripheral NK cells are found in normal quantities and exhibit a distinct phenotype characterized by CD16^hi^CD57^hi^ expression, reduced levels of NK group 2D receptor (NKG2D), low interferon-gamma (IFN-*γ*) production, and elevated intracellular interleukin (IL)-10 [60]. Tumor-infiltrating NK cells, in contrast, show downregulation of CD16, CD57, DNAX accessory molecule-1 (DNAM-1), and NK cell p30-related proteins (NKP30), all of which impair their cytotoxic functions. Interestingly, CD56brightCD16-/dim NK cells, which represent a less cytotoxic phenotype but are capable of releasing IFN-γ when co-cultured with PDAC cells, have been identified as a unique phenotype. Notably, these CD56brightCD16-/dim NK cells were recognized as a positive predictive marker for response to ipilimumab [87] (Figure 2).

In this figure, the distinct phenotypes of NK cells in PDAC are illustrated. Peripheral NK cells are characterized by high expression of CD16^hi^CD57^high^, reduced NKG2D receptor levels, low IFN-*γ* production, and elevated intracellular IL-10. Tumor-infiltrating NK cells exhibit further downregulation of key markers, such as CD16, CD57, DNAM-1, and NKP30, impairing their cytotoxic activity. Additionally, the less cytotoxic CD56^bright^CD16^−/dim^ NK cell subset is identified as the predominant population within Ipilimumab-responsive PDAC tumors, highlighting the importance of phenotyping as a predictive marker.

For abbreviation definitions, please see the legend of Figure 1.

### 2.1. Tumor Characteristics and Their Impact on NK Cell Function

The molecular heterogeneity of PDAC plays a major role in immune evasion, tumor progression, and resistance to treatment [3]. Key mutations, including Kirsten rat sarcoma viral oncogene homolog (KRAS) activation and the inactivation of tumor suppressors like cyclin-dependent kinase inhibitor 2A (CDKN2A), tumor protein p53 (p53), suppressor of mothers against decapentaplegic (SMAD4), phosphatase and tensin homolog (PTEN), deleted in pancreatic cancer-4 (DPC4), myelocytomatosis oncogene (MYC), and breast cancer gene (BRCA), significantly contribute to these processes [88].

While these mutations promote cancer progression and metastasis, they also suppress NK cell activity, which is crucial for anti-tumor immunity. Among these, alterations in PTEN, MYC, p53 and KRAS have the most pronounced effects on NK cell function, though SMAD4 indirectly impacts NK cell activity by promoting EMT [89]. PTEN is a tumor suppressor whose normal expression preserves NK cell activity. However, its overexpression impairs the cytolytic function of NK cells by disrupting the formation of the immune synapses [63]. MYC impairs NK cell maturation by repressing the transcription of signal transducers and activators of transcription (STAT1/2) and reducing Type I interferon (IFN) secretion [67]. p53 normally enhances innate immunity by cross-talking with nuclear factor-kappa B (NF-κB) to eliminate virally infected cells, as seen with the p53 homolog Lvp53 in shrimp. However, the loss of p53 weakens this immune barrier, contributing to tumorigenesis through mechanisms like reactive oxygen species (ROS) induction and toll-like receptor (TLR) activation following infection [90,91]. KRAS mutations, frequently observed in PDAC, activate pathways such as sonic hedgehog (Shh) and NF-κB. Studies in mutant KRAS PDAC models and BxPC-3 cells demonstrate significant pathway activation, while silencing KRAS reduces this activity, underscoring its central role. These pathways not only support tumor growth but also suppress NK cell function. Furthermore, KRAS mutations combined with lifestyle factors, such as obesity, exacerbate NK cell suppression by increasing interleukin-6 (IL-6) production and decreasing interferon-gamma (IFN-γ) levels, impairing NK cell-mediated immune responses. Preclinical in vitro studies reveal that inhibiting KRAS sensitizes PDAC cells to NK cell-mediated attacks, improving anti-tumor NK cell function [64,92,93,94,95,96].

In addition, cluster of differentiation (CD)155, an adhesion molecule highly expressed in PDAC tissues, further impedes NK cell function by reducing the presence of CD226^+^ and CD96^+^ NK cells, thereby promoting immune escape [97]. Enzymes involved in nicotinamide adenine dinucleotide (NAD+) metabolism, such as nicotinamide phosphoribosyltransferase (NAMPT), also contribute to NK cell inhibition and chemoresistance, further complicating treatment responses [68].

Epigenetic plasticity in PDAC, including DNA methylation, has been identified as a significant factor influencing NK cell function and serves as a prognostic markers for PDAC [98]. This plasticity contributes to PDAC heterogeneity, enabling tumor cells to transition into EMT or heterogeneous phenotypes, thereby enhancing metastatic potential and promoting treatment resistance [99]. Specific examples include the hypermethylation of neuronal pentraxin (**NPTX2**) and secreted protein acidic and cysteine rich genes (**SPARC**) in circulating free DNA (cfDNA), which leads to their downregulation and has been associated with PDAC diagnosis and poor prognosis [99,100]. Similarly, the promoter methylation and subsequent silencing of genes such as basonuclin zinc finger protein 1 (**BNC1**) and a disintegrin and metalloproteinase with thrombospondin motif genes (**ADAMTS**) have been identified as promising cfDNA biomarkers for early detection of PDAC [101]. In addition to DNA methylation, histone modifications and non-coding RNAs, such as microRNAs, also play critical roles. For instance, PDAC patients show a significant upregulation of serum microRNAs, including **miRNA-21**, **miRNA-155**, **miRNA-210**, and **miRNA-196a**, which are linked to tumor progression and immune evasion [102]. These epigenetic alterations collectively underscore the complexity of PDAC and its interaction with immune cells like NK cells, highlighting opportunities for biomarker development and targeted therapies.

Transcription factors like GATA-binding factor 6 (GATA6) and pathways such as transforming growth factor-beta (TGF-β) signaling are associated with poor prognosis and impaired NK cell activity, particularly in GATA-low PDAC [51]. GATA6 overexpression also leads to the downregulation of stemness factors such as CD133, aldehyde dehydrogenase gene (ALDH), and SRY-box transcription factor 9 gene (SOX9), leading to increased NK cell activity [52]. In contrast, One Cut Homeobox 3 (ONECUT3) reprograms somatic cells to acquire stemness characteristics, further inhibiting NK cell function and promoting immune evasion [22].

The gut microbiome plays a crucial role in PDAC progression, particularly through its interaction with intratumor microbiome and NK cells. Gut microbiota modulates intratumoral NK cell infiltration and activity, influencing immune responses and tumor progression. Microbial species such as Porphyromonas gingivalis and Fusobacterium nucleatum contribute to cancer growth, while metabolites like trimethylamine N-oxide (TMAO) and lipopolysaccharide (LPS) further impact antitumor immunity by altering NK cell functionality [103,104]. The microbiota, particularly Gammaproteobacteria in PDAC, has been shown to contribute to chemoresistance by inactivating gemcitabine [104]. This class of bacteria could influence immune regulation via cytidine deaminase activity, potentially affecting NK cell activity. However, this interaction requires further investigation to clarify the mechanisms involved and its implications for immune modulation [105]. Dambuza et al. demonstrated that Malassezia globosa (a yeast species from the genus Malassezia and phylum Ascomycota) acts as a carcinogen in PDAC. It promotes tumor-favoring immunity by suppressing NK cell activity through the release of complement factors C3a and C5a, leading to a reduction in NKG2D expression [106].

Furthermore, PDAC cells exhibit significant metabolic plasticity, primarily through glycolytic pathways (Warburg effect), which not only foster chemoresistance but also suppress NK cell activity [107]. Calcium signaling, via purinergic receptor 7 (P2X7R) and related pathways, also plays a critical role in NK cell dysfunction and tumor survival [108,109].

### 2.2. TME Changes and Their Impact on NK Cell Function

The TME plays a pivotal role in the development and progression of PDAC. It is a complex and dynamic environment, composed of cellular stroma, extracellular matrix (ECM) components, and various soluble factors that interact to influence tumor behavior. Within this environment, specific biochemical and cellular mechanisms such as stress and metabolic signaling have a profound impact on NK cells. These mechanisms not only suppress NK cell cytotoxicity but also hinder their ability to proliferate and infiltrate the tumor [3].

#### 2.2.1. Cellular Stroma–NK Cell Interaction

An overview of the cellular components of the PDAC TME and their impact on NK cell function is essential for understanding the immune landscape of pancreatic cancer. The cellular stroma, rich in pancreatic stellate cells (PSCs), cancer-associated fibroblasts (CAFs), and myeloid-derived suppressor cells (MDSCs), creates a complex microenvironment that profoundly influences NK cell activity [18]. PSCs, when activated by cytokines like transforming growth factor beta (TGF-*β*), promote fibrosis and create a dense, rigid (desmoplastic) tumor stroma that impairs NK cell infiltration [73,77]. Notably, PSCs can differentiate into activated PSCs (aPSCs), myofibroblast-like cells that function as CAFs. These aPSCs play a critical role in evading immune surveillance, thereby promoting tumor progression in PDAC patients [110]. A preclinical in vitro study explored whether aPSCs, abundant in the PDAC TME, affect NK cell function. Tumor-derived PSCs from PDAC patients exhibited an activated phenotype and were co-cultured with NK92 cells. The results showed significant suppression of NK cell function-associated proteins (IFN-γ, granzyme B, and CD107a) by aPSCs compared to quiescent PSCs or no co-cultured cells. This suggests that aPSCs in the tumor stroma suppress NK cell activity, contributing to immune suppression in PDAC [111]. Further research aims to uncover the mechanisms of NK cell suppression and advance immunotherapy. For instance, a novel tumor-immune microenvironment (TIME)-on-chip model featuring PANC-1 tumor spheroids, aPSCs, and NK-92 cells provides a valuable platform to study NK cell dysregulation and explore strategies to restore their function in the TME [112].

In addition, CAFs can secrete signaling molecules that inhibit NK cells by downregulating key factors such as IFN-*γ*, perforin, and granzyme or by downregulating key activating receptors like NKG2D and DNAM-1 [29,49]. In some cases, particularly in PDAC with low desmoplasia, the metabolic state of CAFs can further suppress NK cell function. For example, metabolic reprogramming in the stroma may lead to an immune- suppressive environment, where NK cells are less effective in killing tumor cells [29,68].

The upregulation of focal adhesion kinase (FAK) in CAFs, whether through gene amplification or mRNA upregulation, has been associated with reduced NK cell activity, further complicating the immune landscape of PDAC [49]. FAK and steroid receptor coactivator (FAK/Src) signaling pathways have also been identified as key regulators of NK cell suppression [50]. The activation of these pathways in cellular stroma can exacerbate NK cell dysfunction, highlighting their critical role in shaping immune responses in PDAC.

MDSCs suppress NK cell function through various mediators, including TGFβ, indoleamine-2,3-dioxygenase (IDO), and adenosine. TGFβ is produced by MDSCs or MDSC-like cells from prostaglandin E2 (PGE2)-exposed monocytes, while IDO is generated by specific MDSC subsets. TGF-β can downregulate E-cadherin (the major determinant of the epithelial phenotype), which in turn activates histone deacetylase (HDAC1 and HDAC2), leading to NK cell suppression [113]. Adenosine from CD39^high^CD73^high^ MDSCs also inhibits NK cells. These factors reduce NK cell markers such as NKG2D, natural cytotoxicity receptor (NCRs), IFNγ, tumor Necrosis Factor-alpha (TNFα), perforin, granzyme, and ADCC, thereby impairing NK cell activity [114]. Research has demonstrated that co-culturing MDSCs with NK cells leads to a reduction in NK cell-mediated tumor cell cytotoxicity and the induction of immunotolerance. Furthermore, phosphodiesterase-5 (PDE-5) inhibitors hold immunotherapeutic potential by targeting surgery-induced MDSCs, which can restore NK cell function during the clinically relevant perioperative period [115].

NK cell exhaustion, a hallmark of impaired immune function in PDAC, is characterized by the loss of activating receptors such as NKG2D and the upregulation of inhibitory receptors like programmed cell death protein 1 (PD-1), T-cell immunoglobin and mucin domain containing 3 receptor (TIM-3), NKG2A, T-cell immunoreceptor with Ig and ITIM domain (TIGIT), and lymphocyte-activation gene 3 (LAG-3) [71]. Additionally, the proteolytic shedding of major histocompatibility complex class I (MICA/B) proteins, which normally engage activating NK cell receptors, further exacerbates NK cell dysfunction [65]. Within the TME of PDAC, CD11b+ NK cells represent a distinct subset that can contribute to both immune surveillance and immune evasion, depending on their activation state. The balance between these opposing functions plays a critical role in shaping the overall immune response to PDAC [29].

The roles of chemokine signaling on NK cell trafficking are crucial for understanding NK cell function in the TME. Chemokine signaling (e.g., Chemokine (C-X-C motif) receptor 2 (CXCR2) and IL-1) in PDAC patients can induce NK cell migration to tumor sites [6,40]. The downregulation of CXCR2 on NK cells, coupled with CXCR4 overexpression in PDAC, further promotes NK cell suppression and contributes to treatment resistance [37,41]. Targeting CXCR2 in PDAC has produced mixed results, highlighting the need to consider the entire CXCR/CXCL axis and specific chemokines directly involved in activating the receptor. Additionally, its interactions with other cytokines play a critical role and warrant careful evaluation.

The contribution of epigenetic modifications and transcription factors to NK cell dysfunction underscores the intricate relationship between the TME and immune evasion. In particular, histone deacetylase (HDAC) activity and changes in glycogen synthase kinase 3-beta (GSK3B) have been identified as key regulators of NK cell cytotoxicity [53,116]. Wright et al. found that HDAC1 activation in PDAC promotes resistance pathways like EMT, cell cycle regulation, and apoptosis, contributing to chemoresistance. By analyzing genomic and biochemical data, the study identified specific HDAC1 target genes related to GTPase activity and linked them to patient survival. A nine-transcript signature was developed to predict prognosis, highlighting the need for novel treatment strategies in PDAC [66,117].

Transcription factors, such as GATA6 and TGF-*β* signaling, are associated with prognosis in PDAC. In GATA6-low PDAC, their associated epigenetic changes contribute to impaired NK cell function. Conversely, GATA6 overexpression leads to the downregulation of stemness markers (e.g., CD133, ALDH, SOX9), which further enhances NK cell activity [51,52]. Other transcription factors, such as Cbl proteins, NF-*κ*B, Runt-related transcription factor 3 (RUNX3), and STAT, also modulate NK cell function, underscoring the interplay between transcriptional regulation and immune responses in PDAC [26,116,118,119]. Similarly, Homebox A9 (HOXA9) and forkhead box transcription factors (FOXO) have been found to alter NK cell function [120,121,122].

Additionally, the effects of tumor angiogenesis and miRNAs on NK cell activity within the TME are critical factors influencing immune function in PDAC. Tumor angiogenesis in PDAC, involving NK cells, has been shown to affect immune function. For instance, STAT5-deficient NK cells overproduce VEGF-A, leading to a reduction in NK cell cytotoxicity [123]. MiRNAs, including both oncogenic (oncomiRs) and tumor-suppressive (tsmiRs) miRNAs, impact pathways like phosphoinositide-3-kinase (PI3K), contributing to PDAC aggressiveness and NK cell dysfunction [124,125,126,127].

Metabolic modulators and metabolic reprogramming within the TME also have a significant impact on NK cell function in PDAC. The IDO enzyme, produced by immunoregulatory cells, inhibits the expression of key NK cell receptors such as NKG2D and NKp46, impairing NK cell activity through STAT signaling pathways in PDAC [55]. Additionally, NAD+-related proteins like SIRT1 and the deacetylation of FOXO are other crucial metabolic modulators that alter NK cell function [128]. Metabolic reprogramming within the TME also affects NK cell activity. Lipid metabolism and high-fat diets, with signaling pathways like CCK and PPAR, contribute to NK cell dysfunction. Lipid droplets (LDs) are organelles that store fats and are involved in energy metabolism. In PDAC, LDs are increased and linked to tumor growth. Pancreatic CSCs have more LDs, and activating PPARα, a key factor in LD signaling, may help maintain CSC properties [129]. Reducing LDs limits invasion in KRAS-mutant PDAC [130], which may help decrease immune suppression, tumor invasion, and drug resistance [27,131]. Together, these metabolic changes create an immune-suppressive environment that further hinders NK cell-mediated anti-tumor responses [27,28,132,133,134,135].

Stress and calcium signaling are other critical factors influencing NK cell function in PDAC as well. Stress-induced signaling, such as the expression of MIC-A/B and UL 16-binding proteins (ULBPs), impairs NK cell activity through IL-6 and STAT3 signaling pathways. Elevated IL-6 levels in PDAC are particularly concerning, as they are associated with reduced NK cell-mediated IFN-γ secretion, which contributes to poor prognosis in PDAC patients [61,85]. Hypoxia is another key factor that alters NK cell function; under low oxygen conditions, hypoxia-inducible factor 1 alpha (HIF-1α) is upregulated, leading to the downregulation of activating NK receptors such as NKG2D, NKp30, NKp44, and NKp46, thereby promoting NK cell suppression [86]. Additionally, the expression of CD73 in response to hypoxia further inhibits NK cell activity [32]. Calcium signaling, particularly through channels like Orai calcium release-activated calcium modulator 1 (Orai1), stromal interaction molecule-1(STIM1), and purinergic receptor 7 (P2X7), plays a role in chemotherapy resistance, with PSCs secreting IL-6 to increase store-operated calcium entry (SOCE), which further suppresses NK cell activity and promoting PDAC aggressiveness [74,75,136].

This structure allows for a more holistic understanding of how diverse stromal components and molecular pathways contribute to NK cell suppression and immune resistance in PDAC.

#### 2.2.2. Extracellular Matrix (ECM)–NK Cell Interaction

The ECM plays a critical role in shaping the progression of PDAC and influencing NK cell function. Key ECM components such as collagen, proteoglycans, elastin, and integrins (e.g., *α*2*β*1 and *α*V*β*3) are essential not only in tumor development but also in modulating NK cell behavior [45,137,138]. One study identified four integrin genes (ITGB1, ITGA5, ITGA6, and ITGA2), which were overexpressed in PDAC, linking to poorer patient survival [139]. The overexpression of these integrins in PDAC could impair NK cell infiltration, migration, and cytotoxic activity by remodeling the ECM [140].

One of the major ECM remodeling mechanisms in PDAC involves matrix metalloproteinases (MMPs), which degrade ECM components, facilitating tumor cell invasion and migration. This remodeling of the ECM can also affect NK cell activation and has been identified as a potential predictive marker for PDAC prognosis [141,142]. A preclinical study demonstrated that MMP-9 overexpression led to reduced expression of NK cell activating receptors (NKG2D, DNAM-1, NKp30, NKp46), lower production of perforin and granzyme B, and decreased secretion of TNF-α and IFN-γ. Inhibiting MMP-9 was shown to restore NK cell activity [55].

Signaling pathways within the ECM contribute significantly to immune suppression in PDAC. Hyaluronic acid (HA), Sonic Hedgehog (Shh), and Src/FAK signaling play important roles in desmoplasia, hypoxia, and angiogenesis, creating an immunosuppressive microenvironment that enhances PDAC progression and drug resistance [143,144]. The Hedgehog signaling pathway, also activated by KRAS, further suppresses NK cell activity through its effectors, the Gli-family transcription factors. Scales et al. demonstrated that loss of Gli2 and Gli3 reduces tumor growth in PDAC by recruiting NK cells. Gli2 and Gli3 are key mediators of Hedgehog signaling that regulate various cellular processes, including tumor–stroma interactions and immune modulation. NK cell depletion reversed the tumor-suppressive effect of Gli2/Gli3 loss, restoring tumor growth. This indicates that combined Gli2/Gli3 deletion in fibroblasts suppresses tumor growth specifically through enhanced NK cell recruitment [96].

Furthermore, the Src/FAK pathway, activated by growth factors such as epidermal growth factor (EGFR), vascular endothelial growth factor (VEGF), fibroblast growth factor (FGF), and platelet-derived growth factor (PDGF), enhances tumor cell survival and immune escape [80]. Another important signaling mechanism in PDAC is TGF*β* signaling, which acts at both the cellular and ECM levels and is implicated in promoting immune suppression and treatment resistance. Although targeting TGFβ and Shh pathways has shown promise in preclinical studies, clinical application remains controversial due to mixed results [145].

In addition to these signaling pathways, stress-induced mechanisms such as the creation of a fibrotic stroma, hypovascularization, and immunosuppression further complicate treatment resistance in PDAC. The ECM environment, in combination with these stress factors, impairs NK cell function and limits the effectiveness of therapies [146]. For instance, Death-Associated Protein 1 (DAP1), a modulator of autophagy, has been linked to treatment resistance in PDAC. DAP1 expression is low in several malignancies, including PDAC, and its potential role in influencing NK cell functionality and therapy resistance is an emerging area of research [42]. A clinical study using in vitro analysis found that DAP3 was significantly overexpressed in pancreatic tumor tissues, while DAP1 was downregulated. Elevated DAP3 levels were associated with shorter survival and worse prognosis, especially when combined with lymph node involvement [43].

**Figure 3:**
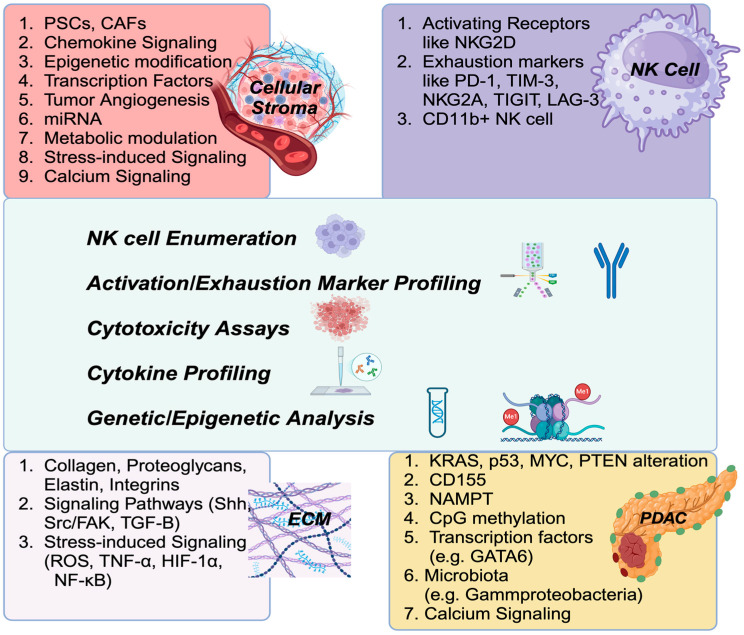
NK cell diagnostic application.

The diagnostic approach to assessing NK cell function provides crucial insights into the immune landscape of cancer, particularly PDAC. Diagnostic approaches can provide valuable insights into NK cell activity within the TME. Understanding the etiology of NK cell suppression in the context of PDAC, particularly focusing on tumor characteristics and TME changes, is essential for designing the most effective diagnostic approaches (Figure 3). This knowledge helps identify immune dysfunction and guides the selection of NK cell-related biomarkers for accurate prognosis and therapeutic decisions. It also aids in the development of personalized immunotherapies, such as NK cell-based treatments and immune checkpoint inhibitors, and supports monitoring treatment responses, ultimately improving patient outcomes and advancing precision oncology.

This figure outlines the diagnostic strategies for assessing NK cell function in the context of cancer, particularly PDAC. Approaches such as NK cell enumeration, profiling of activation and exhaustion markers, cytotoxicity assays, cytokine profiling, and genetic/epigenetic analyses provide essential information on NK cell activity within the tumor microenvironment (TME). ***Upper left section***: Cellular Stroma. Some specific markers related to cellular stroma of the TME; ***Lower left section*:** ECM (Extra Cellular Matrix). Some specific markers related to extracellular matrix of the TME; ***Upper right section*:** NK cells. Some specific markers related to natural killer cells. ***Lower right section***: PDAC. Some specific markers related to tumor cells. For abbreviation definitions, please see the legend of Figure 1.

## 3. NK Cells: Therapeutic Potential

**Figure 4:**
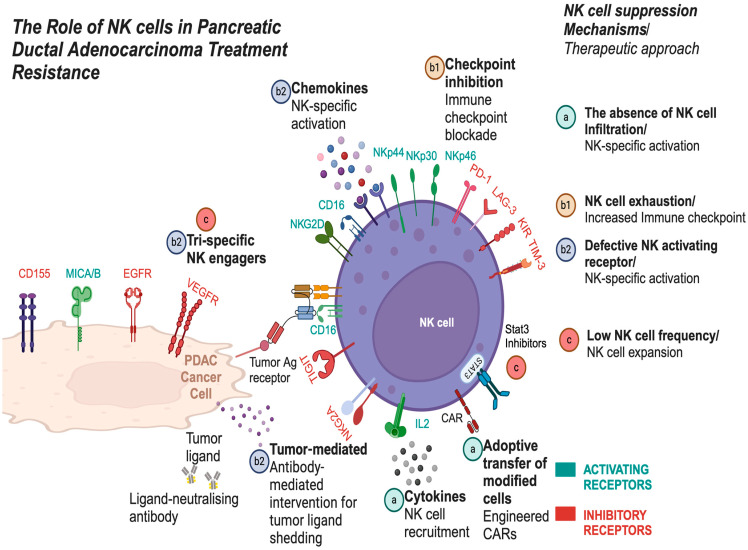
Targeting NK cell suppression.

**Figure 5:**
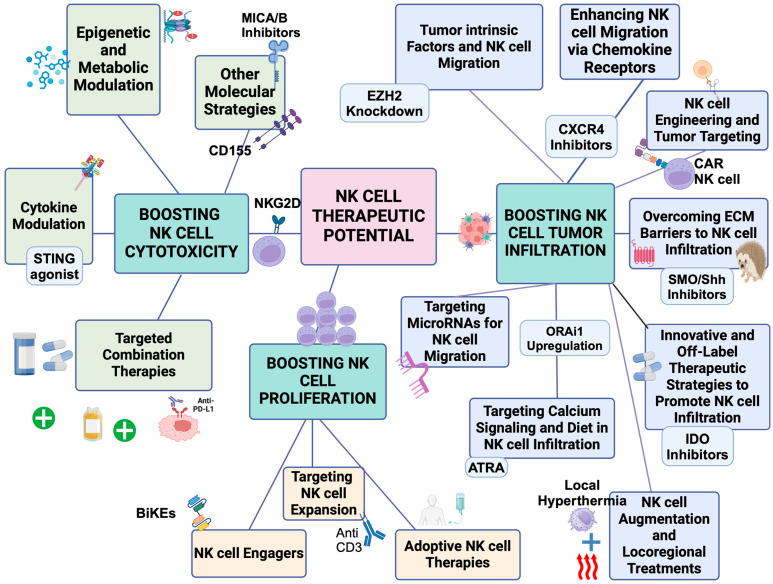
NK cell therapeutic application.

Understanding PDAC treatment resistance is crucial, especially when addressing the main suppressive mechanisms within tumor and the TME that limit NK cell function. These include reduced NK cell cytotoxicity, impaired proliferation, and limited tumor infiltration (Figure 4, Table 2). Efforts to counter these suppressive factors have led to various therapeutic strategies (Figure 5) targeting tumor cells (including cancer stem cells), ECM remodeling, and enhancing NK cell activity in PDAC [147,148]. Enhancing NK cell cytotoxicity, increasing NK cell frequency, and improving tumor infiltration are pivotal strategies for optimizing therapeutic outcomes in PDAC. Notably, chemotherapy (e.g., gemcitabine) has shown potential in increasing NK cell activity [149], but the challenge remains to overcome resistance and optimize NK cell function within the PDAC TME. Understanding how these strategies impact the phenotypic subtypes of PDAC (E/QM states) is crucial. For example, FOLFIRINOX, one of the most effective therapies for PDAC, can initially provide significant therapeutic benefits. However, over time, it may drive tumor subtypes toward a more immunosuppressive mesenchymal state, ultimately reducing NK cell activity and contributing to treatment resistance. In contrast, therapies like vitamin D have shown promise in reducing EMT, enhancing NK cell function and potentially mitigating some of the immunosuppressive effects within the TME [24,150].

This figure illustrates the three main mechanisms by which NK cell activity is suppressed—low NK cell proliferation, tumor infiltration, and NK cell cytotoxicity—along with strategies to address each of these suppression mechanisms: (**a**) NK cell tumor infiltration defects: The first mechanism depicts impaired NK cell infiltration into tumors. Strategies to overcome this include NK cell recruitment and adoptive transfer using CAR NK cells. (**b1**) NK cell exhaustion: NK cell exhaustion is characterized by the upregulation of immune checkpoint molecules like PD-1, which reduce NK cell cytotoxicity. Immunotherapies, such as checkpoint inhibitors, can help reverse this exhaustion. (**b2**) Defective receptor signaling: This mechanism addresses defective receptor signaling. Blockade of inhibitory receptors (e.g., KIRs, NKG2A) and activation of NK cell receptors using NK cell engagers can enhance NK cell function. (**c**) Low NK cell frequency: The third mechanism illustrates diminished NK cell abundance. This can be targeted by STAT3 inhibitors, which promote NK cell expansion. Additionally, NK cell engagers can be used to stimulate NK cell proliferation, further enhancing their presence and activity. For abbreviation definitions, please see the legend of Figure 1.

This figure outlines various therapeutic strategies to enhance NK cell function in cancer treatment, categorized into three key goals: Boosting NK cell cytotoxicity, boosting NK cell proliferation, and promoting NK cell tumor infiltration.

***Left section:*** Boosting NK cell cytotoxicity. Strategies focus on enhancing NK cell-killing activity, including epigenetic and metabolic modulation, cytokine modulation, immune checkpoint inhibition, targeted combination therapies, and immune receptor signaling pathway targeting to activate NK cells and improve tumor response.

***Middle section***: Boosting NK cell proliferation. Approaches aimed at expanding NK cell numbers, such as NK cell expansion, NK cell engagers, and allogeneic NK cell therapies, to strengthen immune responses.

***Right section:*** Promoting NK cell tumor infiltration. Methods to enhance NK cell migration into tumors, including targeting tumor-intrinsic factors, chemokine receptor modulation, NK cell engineering, overcoming ECM barriers, and exploring innovative therapies to improve NK cell infiltration and effectiveness. For abbreviation definitions, please see the legend of Figure 1.

### 3.1. Boosting NK Cell Cytotoxicity

Enhancing NK cell cytotoxicity is central to overcoming immune suppression in PDAC. Several therapeutic strategies aim to activate NK cells and bypass immunosuppressive barriers, improving their ability to eliminate tumor cells. Key strategies include epigenetic modulation, metabolic modulation, cytokine modulation, and targeting immune checkpoints.

#### 3.1.1. Epigenetic and Metabolic Modulation

Targeting EZH2, a histone methyltransferase, can enhance NK cell activity in PDAC by reversing immune suppression. EZH2 promotes EMT by interacting with SNAI1 (Snail), down-regulating E-cadherin and suppressing NK cell function. Inhibiting EZH2 restores NK cell activity and enhances their ability to kill PDAC cells [48]. Moreover, histone acetyltransferase (HAT) inhibitors (e.g., curcumin), HDACs blockers, and IDH1/2 inhibitors are being explored for their potential to enhance NK cell-mediated cytotoxicity in PDAC [54]. These epigenetic modulators offer a potential avenue for activating NK cells and improving immune responses in PDAC. Preclinical studies have indicated that simultaneous inhibition of GSK3B and HDACs can enhance gemcitabine-induced apoptosis by NK cells [53]. HDACs can also suppress the expression of NKG2D ligands; supporting this, a study investigating the combined use of valproic acid, an HDAC inhibitor, and gemcitabine in PDAC found that this combination can increase NKG2D ligand expression, thereby enhancing the immune response [66].

In addition, metabolic modulation has emerged as a promising strategy for enhancing NK cell cytotoxicity in PDAC. NAD + supplementation, combined with STING agonists and type I interferon signaling, activate NAD-consuming enzymes and enhances PDAC sensitivity to therapies like NAMPT inhibitors, boosting NK cell function [69]. Modifying the NAD+/NADH ratio can also induce apoptosis and enhance the effects of metformin in PDAC by targeting cancer stem cells (CSCs) [200]. Metformin has shown potential in enhancing NK cell cytotoxicity in other cancers [151], and in PDAC. Sancho et al. used metformin as a mitochondrial-targeted agent to target PDAC CSCs, demonstrating that targeting MYC can sensitize resistant CSCs to metformin [148].

#### 3.1.2. Cytokine Modulation

Cytokines play a critical role in regulating NK cell function and modulating the immune response in PDAC. IL-12 has been shown to enhance NK cell cytotoxicity, while blockading IL-10 can reduce NK cell suppression and boost antitumor immunity in PDAC and other tumors [62,152,153]. A clinical trial demonstrated the anti-tumor activity of PEGylated human IL-10 (AM0010) in patients with pancreatic cancer. Preliminary results showed promising clinical outcomes, and mechanistic data revealed enhanced immune stimulation, supporting further investigation of AM0010 [152]. MCBD-IL12, a bioengineered IL-12 therapy, suppresses liver metastases in murine PDAC models by activating NK cells, showing promise for treating metastatic PDAC [154]. Combination therapies using cytokines for enhanced NK cell cytotoxicity have shown promise in boosting NK cell cytotoxicity in PDAC. For instance, combining cetuximab with IL-21 has significantly enhanced NK cell activity in EGFR-positive PDAC, regardless of KRAS mutation status [47].

In addition, in poorly differentiated high-grade PDAC, particularly with a mesenchymal subtype, elevated secretion of colony-stimulating factor 1 (CSF-1) promotes the differentiation of monocytes into anti-inflammatory M2-like tumor-associated macrophages (TAMs). These M2-like TAMs facilitate tumor progression and immune suppression [34]. CSF1/1R inhibitors reprogram TAMs from an immunosuppressive to a pro-inflammatory phenotype, boosting NK cell activation through cytokines like IL-1β, IFN-γ, and IL-15. However, blocking CSF-1R may reduce NK cell numbers by depleting IL-15-producing myeloid cells. Although clinical trials in PDAC show limited efficacy, TAM modulation holds potential in combination immunotherapy [35,57,155,156].

Additionally, the STING pathway has been identified as a potential target for enhancing NK cell function. Activation of the STING pathway, particularly using STING agonists like DMXAA, has been shown to promote type I interferon (IFN-I) responses and improve the antitumor properties of NK cells. In combination with gemcitabine, STING agonists may stimulate the immune system by promoting inflammatory cytokine production, further enhancing the immune response against PDAC [80,81].

Another strategy to boost NK cell function in PDAC is the use of combining NK cells with TGF-β neutralizers. TGF-β is an immunosuppressive cytokine that often contributes to immune evasion in the TME. By neutralizing TGF-β, the immune response can be enhanced, and NK cell activity can be restored, promoting better tumor elimination [58,145].

A promising approach to cytokine modulation involves cytokine-armed vaccines, such as cytokine-armed vaccinia viruses (vvDD-IL2 and vvDD-IL15), which have shown great potential in activating NK cells in difficult-to-treat cancers like PDAC. These viruses trigger immunogenic cell death (ICD) in tumor cells, releasing danger signals that activate NK cells, leading to enhanced cytotoxicity. In preclinical studies, co-culturing virus infected PDAC cells with NK cells resulted in the activation of a rare NK subpopulation (CD56^dim^ CD16^dim^) and increased tumor killing, primarily through Fas ligand interactions. This demonstrates how cytokine modulation via virus-mediated vaccines can not only activate NK cells but also enhance their anti-tumor response, offering a novel strategy for cancer immunotherapy [157].

#### 3.1.3. Targeted Combination Therapies

Targeted combination therapies aim to enhance NK cell cytotoxicity and improve tumor elimination in PDAC by utilizing multiple modalities. The coordinated activation of NKG2D, DNAM-1, and natural cytotoxicity receptors (NCRs) plays a significant role in NK cell-mediated elimination of PDAC tumor cells [6]. One particularly intriguing modality that can be combined with conventional PDAC therapy, off-label approaches, or other innovative treatments is targeting STAT3 in NKp46+ cells. This approach has been shown to boost NK cell cytotoxicity, offering a promising therapeutic option [170]. STAT3 activation in NK cells reduces their effectiveness by downregulating essential receptors such as NKG2D, NKp30, and DNAM-1, while also decreasing the production of perforin and granzyme B. This impairs their ability to kill tumor cells. In addition to these intrinsic effects, STAT3 activation within the TME exacerbates immunosuppression by reducing NK cell recruitment and downregulating the expression of ligands required for tumor cell recognition and destruction [201]. Similarly, targeting TIGIT signaling is crucial, particularly in conjunction with neoantigen vaccines. Neoantigen vaccines in PDAC are limited by immune suppression mechanisms, including high expression of TIGIT ligands like CD155 in tumors. TIGIT signaling impacts immune function, including NK cells, which also express TIGIT and can be suppressed in the TME. Combining neoantigen vaccines with TIGIT blockade may enhance NK cell-mediated anti-tumor responses alongside T-cell effects. Overall, findings from both preclinical and human studies suggest that combining neoantigen vaccines with treatments targeting the TIGIT signaling pathways holds promise for improving outcomes in patients with PDAC [82].

Locoregional and inhibitory interventions such as FAK inhibition, are increasingly being explored as methods to counteract the immunosuppressive and fibrotic characteristics of the PDAC TME [202]. In an animal model of PDAC, a combination of checkpoint immunotherapy, radiotherapy, and FAK inhibition led to a complete response and long-term survival [158]. FAK inhibitors boost immune surveillance by reversing the fibrotic, immunosuppressive properties of the PDAC TME, thus increasing the tumors’ responsiveness to immunotherapy. Notably, FAK inhibition has been shown to enhance the sensitivity of PDAC cells to radiation in laboratory settings [159], providing a promising prospective therapeutic approach for clinical use.

Targeting immune receptors including immune checkpoint inhibitors, combined with other treatments, has also shown promise in overcoming the immunosuppressive TME in PDAC. For example, the PD-L1 inhibitor durvalumab, when combined with the CD40 agonist sotigalimab and gemcitabine/nano-albumin-bound paclitaxel, has been shown to enhance NK cell-mediated tumor destruction and improve overall treatment efficacy [160]. By targeting PD-L1, durvalumab helps reverse immune suppression within the TME, enabling NK cells and other immune cells to better target and eliminate cancer cells. In addition to PD-L1, other immune checkpoint molecules such as TIM-3 and TIGIT are also being explored as potential targets to further enhance NK cell cytotoxicity and improve therapeutic outcomes [83,84].

Another strategic approach involves using PD-1 checkpoint inhibitors to prevent NK cell exhaustion, thereby enhancing their antitumor activity in PDAC [203]. This approach can, for example, be considered if resistance occurs during conventional treatment and exhaustive markers can be identified. Clinical trials are currently evaluating combinations of VEGFR inhibitors with anti-PD-1 therapies in solid tumors, including PDAC (ClinicalTrials.gov identifiers: NCT03797326, NCT04887805) [76]. Additionally, a case report showed a complete response in a heavily pretreated PDAC patient following treatment with a combination of lenvatinib and pembrolizumab. These results underscore the potential of checkpoint inhibitors, like those targeting PD-1/PD-L1 and TIM-3, in restoring NK cell function and improving tumor elimination [83,84,161].

Moreover, targeting signaling pathways, such as pyruvate kinase muscle 2 (PKM2), which regulates PD-L1 expression, could further enhance NK cell function and overcome resistance in PDAC [162]. Studies also suggest that combining granulocyte-macrophage colony-stimulating factor gene-transduced autologous pancreatic cancer vaccines (GVAX) with CSF-1R inhibitors and anti-PD-1 therapy can increase IFN-*γ* levels and enhance NK cell cytotoxicity [36]. Additionally, blocking both IL-6 and PD-1 has been shown to increase antitumor activity, likely due to reduced NK cell exhaustion and enhanced NK cell cytotoxicity [61].

#### 3.1.4. Other Molecular Strategies

Several novel molecular strategies are being explored to regulate NK cell activity and enhance immune responses in PDAC. For example, targeting diacylglycerol kinase (DGK) ζ, a negative regulator of NK cell activity, is one of the promising strategies for modulating signaling pathways to enhance NK cell function in PDAC. Recent studies are exploring the use of DGK inhibitors to improve NK cell-mediated tumor elimination [44]. Similarly, post-translational modifications, such as ubiquitination, have been shown to alter NK cell signaling [204]. For instance, Cbl knockdown can decrease Vav protein ubiquitination, leading to increased NK cell- killing activity, highlighting a novel avenue for enhancing NK cell function in PDAC [163].

CD73, a membrane-bound nucleotidase that generates extracellular adenosine, plays a key role in immune suppression. Inhibition of CD73 by diclofenac has shown promising results in managing PDAC metastasis, demonstrating greater efficacy than CD73-blocking antibodies. Furthermore, diclofenac has the potential to enhance the effectiveness of gemcitabine, promoting NK cell activity in PDAC models and possibly improving overall therapeutic outcomes [33].

A preclinical study demonstrated that low-dose gemcitabine treatment leads to increased MICA/B expression, enhancing NK cell function in PDAC, rather than directly promoting cytotoxicity [66]. However, inhibiting the cleavage and release of MIC molecules from the tumor surface could potentially enhance NKG2D-dependent cytotoxicity [65].

Other strategies involve targeting alternative splicing of CD155, which may help overcome immune evasion by tumor cells [31]. Targeting the complement system pathway may provide an additional mechanism to combat drug resistance in PDAC. This strategy could help modulate the immune environment, increasing NK cell activity and tumor cell recognition [205].

Lastly, the use of aptamers, small single-stranded oligonucleotides, may assist in remodeling immune cell phenotypes from a protumor to an antitumor state, enhancing the immune system’s ability to fight cancer [206]. Several preclinical studies [164] and clinical trials are exploring aptamer-based therapies like AS1411 (anti-nucleolin) and Toll-like receptor 4 antagonist aptamer (apTOLL) [165]. Notably, AS1411’s anti-nucleolin activity may disrupt tumor-driven immune evasion by potentially upregulating ligands that activate NK cell receptors, such as NKG2D. While direct studies on apTOLL’s impact on NK cells may be limited, its role in modulating immune responses through TLR4 antagonism could indirectly enhance NK cell function in cancer therapy [165,166]. Furthermore, aptamer-drug conjugates (ApDCs) show promise for challenging cancers like PDACs by overcoming treatment obstacles and enabling precise drug delivery, although they remain in the early stages of development [167]. For instance, aptamer-NK cell assemblies have been reported to enhance targeted cell recognition and promote anti-tumor immunity [168].

### 3.2. Boosting NK Cell Proliferation

Increasing NK cell proliferation has been shown to correlate with improved outcomes in various cancers, including PDAC [207]. Several strategies have been explored to enhance NK cell numbers and their functional activity, particularly in solid tumors like PDAC. These strategies can be categorized into three main approaches: targeting NK cell expansion, using NK cell engagers, and utilizing allogeneic NK cell therapies.

#### 3.2.1. Targeting NK Cell Expansion

One approach to boosting NK cell numbers involves directly modulating their expansion. Several studies have demonstrated the effectiveness of targeting specific molecules to increase NK cell proliferation. For example, antibodies targeting CD3 and CD52 have been shown to increase the abundance of effector NK cells in the peripheral blood of PDAC mouse models [169]. Additionally, knocking out the STAT3 pathway has been shown to increase the proliferation of cytotoxic NK cells, thereby enhancing their tumor-killing capacity [170]. These findings underscore the importance of targeting key signaling pathways to promote NK cell expansion.

#### 3.2.2. NK Cell Engagers (NKCEs)

Another promising strategy for enhancing NK cell proliferation is the use of NK cell engagers, such as bi-specific and tri-specific NK cell engagers (BiKEs and TriKEs). These engineered molecules are designed to bring NK cells near tumor cells, facilitating more efficient tumor-targeting and promoting NK cell proliferation and tumor infiltration [70]. By engaging multiple targets simultaneously, BiKEs and TriKEs not only boost NK cell numbers but also enhance their tumor-killing capacity, making them an exciting therapeutic avenue for PDAC and other cancers. The efficacy of both BiKEs and TriKEs has been demonstrated in vitro and in preclinical models [171]. Laurent et al. introduced a multispecific platform that allows co-engagement of up to three NK cell activating receptors and two tumor antigens, showing stronger anti-tumor activity in preclinical models than standard monoclonal antibodies (mAbs) like rituximab, obinutuzumab, and cetuximab. These results support the clinical development of NKCEs as a complementary approach to current immuno-oncology therapies [70]. Kaminski et al. introduced cam1615TEM8, a novel TriKE molecule that activates NK cells with IL-15 and targets TEM8-positive tumor and stromal cells, offering a multifaceted approach to solid tumor therapy [172]. Notably, a study by Gauthier et al. developed a potent tri-specific NK cell engager (TriKE) to target a tumor-specific antigen and two receptors on NK cells (NKp46 and CD16). By engaging these receptors, TriKEs direct NK cells toward tumor cells, enhancing their ability to target and destroy cancerous cells effectively [70]. Additionally, IL-15/B7-H3 TriKEs-based immunotherapy has shown promising preclinical efficacy for PDAC [173].

#### 3.2.3. Adoptive (Allogeneic and Autologous) NK Cell Therapies

Allogeneic NK cell-based therapies are emerging as a promising approach for treating solid tumors, including PDAC. Several studies have investigated the safety and efficacy of NK cell infusion therapies. For instance, a clinical case study demonstrated the effectiveness and safety of combining NK cell-based immunotherapy with conventional treatments in a patient with advanced PDAC. The patient experienced significant tumor regression and normalized biomarker levels after receiving NK cell-enriched allogeneic lymphocyte infusions alongside chemotherapy and targeted therapy. Mild and manageable side effects were observed, supporting this combined approach as a potential strategy for treating advanced PDAC [174]. Similarly, a preclinical study in a mouse model using allogeneic NK cells with NKG2D and NKp30 receptors showed reduced PDAC tumor growth and improved survival. Notably, no other conventional therapies were used, highlighting the potential of NK cell therapy alone as an effective treatment for PDAC [175]. In another clinical trial, the combination of irreversible electroporation (IRE) and allogeneic NK cell therapy for metastatic PDAC led to improved immune function, reduced tumor markers, and enhanced short-term outcomes and quality of life. This study confirmed the safety and effectiveness of this treatment combination for metastatic PDAC patients [176]. Lastly, a study investigating NK cell therapy combined with chemotherapy found that activated NK cells expanded with autologous peripheral blood mononuclear cells exhibited enhanced cytotoxicity against PDAC cells. In a mouse model, the combination of these NK cells with gemcitabine and erlotinib effectively inhibited tumor growth, suggesting that NK cell therapy, when paired with chemotherapy, could improve treatment outcomes for PDAC [177].

### 3.3. Promoting NK Cell Tumor Infiltration

In PDAC tumors, NK cells are often scarce, accounting for less than 0.5% of the immune cell population, which contributes to the poor prognosis of the disease. Since mutations in the KRAS gene are the earliest genetic variation in PDAC [176] and lead to a loss of NK cells in precancerous stages, several strategies to enhance NK cell tumor infiltration [208] and NK adoptive cell transfer are being explored as promising therapeutic interventions. These strategies target tumor-intrinsic factors, chemokine pathways, and the TME to enhance NK cell infiltration into tumors and overcome therapeutic resistance.

#### 3.3.1. Tumor-Intrinsic Factors and NK Cell Migration

Tumor-intrinsic factors play a critical role in NK cell migration and tumor infiltration. For example, EZH2 knockdown or the combination of EZH2 blockade with senescence-inducing therapies, such as trematinib/palbociclib, has resulted in increased NK cell migration and even complete responses in some PDAC mouse models [46]. Hu et al. utilized adoptive cell therapy in a KRAS and p53-mutated PDAC mouse model and further demonstrated that enhancing NK cell infiltration into tumors led to increased tumor necrosis and prolonged survival in PDAC mouse models, compared to controls [209]. Additionally, this study highlights how tumor-intrinsic mutations, such as KRAS and p53, may shape the microenvironment to influence immune cell behavior, including the recruitment and migration of NK cells.

#### 3.3.2. Enhancing NK Cell Migration via Chemokine Receptors

Modifying the chemokine receptor signaling is another effective strategy to improve NK cell infiltration. For example, CXCR2 ligands or CXCR4 blockade have been shown to enhance NK cell recruitment into PDAC tumors [6,41]. Interferons stimulate the release of CXCL10, which further promotes NK cell migration toward the tumor site [210].

#### 3.3.3. NK Cell Engineering and Tumor Targeting

NK cell engineering also holds promise for improving NK cell infiltration and targeting PDAC cells. One such strategy involves using aptamers to attach and modify NK cells to facilitate targeting toward cancer cells [211]. A clinical trial is exploring the combination of NOX-A12, a CXCL12 antagonist, with pembrolizumab in PDAC to enhance NK cell recruitment and improve immune responses by both directly targeting the tumor-promoting chemokine CXCL12 and releasing the brakes on the immune system. By blocking CXCL12, NOX-A12 aims to disrupt the tumor-promoting microenvironment, hinder cancer progression, and enhance NK cell tumor infiltration [38,125].

Another innovative approach is the use of NK cell-recruiting protein-conjugated antibodies (NRP-bodies). These antibodies can induce NK cell infiltration into tumors by binding to CXCL16, while simultaneously activating Ras Homolog Family Member A (RhoA) through the extracellular signal-regulated kinase (ERK) signaling cascade, further boosting NK cell-mediated tumor targeting [39].

In addition to aptamer-based approaches and NRP-bodies, chimeric antigen receptor (CAR) NK cell therapy has emerged as a promising strategy to improve NK cell specificity and efficacy against PDAC. In a study by Xia et al., anti-Roundabout Guidance Receptor 1 (ROBO1) CAR NK-92 cells were tested in combination with brachytherapy in an orthotopic pancreatic tumor model, leading to superior outcomes compared to brachytherapy alone. This combinatorial approach effectively targeted PDAC cells and enhanced treatment efficacy [78]. Similarly, Froelich et al. demonstrated that PSCA-targeted CAR NK cells significantly improved cytotoxic activity and survival rates in a metastatic humanized PDAC mouse model after 48 days. The reduction in tumor cell numbers, alongside increased NK cell infiltration within the TME, underscores the therapeutic potential of CAR NK cells in PDAC [178]. Moreover, PSCA CAR-S15 NK cell therapy is emerging as a safe and effective NK cell-based immunotherapy option for PDAC [179]. Further advancements include the genetic engineering of CAR NK cells derived from induced pluripotent stem cells (iPSCs), enabling persistent NK cell activation and prolonged antitumor effects in PDAC mouse models. Additionally, STING agonists, such as cGMAP, a signaling molecule associated with immune pathways, in combination with the CAR NK cell, have been suggested as adjuvants to enhance NK cell therapy in PDAC by directly activating NK cell cytotoxicity [180]. In a study focused on PDAC with liver metastasis, CAR NK cell infusion targeting the roundabout guidance receptor 1 (ROBO1) was found to be safe and led to stable disease in patients following five months of weekly infusions [59]. Further supporting this approach, ROBO1 was overexpressed in liver metastatic CK19 + PDAC cells, suggesting that targeting ROBO1 with NK cell therapies could effectively overcome metastatic spread in PDAC [79].

#### 3.3.4. Overcoming ECM Barriers to NK Cell Infiltration

The ECM is a significant barrier to NK cell infiltration in PDAC, and targeting ECM- related resistance mechanisms in PDAC, including desmoplasia, offers a promising approach to combat chemoresistance. By improving ECM remodeling, these strategies can facilitate better penetration of therapeutic agents and enhance NK cell infiltration. Several strategies like the use of MMP inhibitors [212], hyaluronidase, Shh inhibitors, fibroblast activation protein (FAP) targeting agents, and CXCR4 inhibitors have been discussed to manage therapeutic resistance in PDAC [3]. MMP inhibitors degrade ECM components, improving NK cell access to tumor cells and enhancing immune responses [55,141,142]. Hyaluronidase breaks down hyaluronic acid in the ECM, increasing tissue permeability, allowing for better NK cell infiltration, and potentiating the effects of chemotherapy [144]. For example, researchers have engineered a modified Salmonella typhimurium (ST) that expresses bacterial hyaluronidase (bHs-ST), which depletes human hyaluronic acid (HA) in PDAC tumors. Their findings show that bHs-ST targets and colonizes PDAC tumors, depletes HA, and significantly improves ST spread within fibrous tumors [181].

Additionally, PEGPH20, a hyaluronidase, may enhance radiation therapy’s effectiveness in PDAC, but only in tumors with high levels of hyaluronan [182]. Furthermore, inhibiting hedgehog signaling with agents like IPI-269609, a potent hedgehog inhibitor, has shown promise in controlling tumor growth. While it does not improve the efficacy of gemcitabine in PDAC, it effectively controls tumor progression, supporting the idea that its action is primarily through immune activation rather than direct chemotherapy enhancement [183]. In contrast, Saridegib (IPI-926), a newer class of hedgehog inhibitors and a potent smoothened (Smo) receptor antagonist, has been demonstrated to normalize tumor vasculature, improve drug delivery, and indirectly enhance immune cell infiltration [184]. Blocking the Src/FAK pathway with VS-4718 has been shown to decrease the density of the ECM and potentiate the effect of chemotherapy [49,50,158,159,185]. Additionally, vitamin D analogs have been shown to increase the concentration and efficacy of gemcitabine by reducing tumor-associated fibrosis and increasing NK cell infiltration [30,186]. Interestingly, all-trans retinoic acid (ATRA), an active metabolite of vitamin A, can alter PSCs, potentially preventing matrix remodeling and reducing cancer cell invasion, thereby indirectly supporting NK cell tumor infiltration [77].

In addition, by targeting molecules like BRD4 and CD11b, we can modulate the TME, which helps address barriers like desmoplasia (fibrosis) and other ECM-related factors that restrict immune cell infiltration. CD11b agonists reconfigure innate immunity, making PDAC more responsive to immunotherapies [213]. Preclinical studies have also demonstrated that targeting BRD4 with bromodomain inhibitors can not only affect tumor growth but also reshape the ECM, facilitating NK cell migration and infiltration, especially in metastasis-prone areas like the liver [56].

#### 3.3.5. Innovative and Off-Label Therapeutic Strategies to Promote NK Cell Infiltration

Innovative therapies are also being explored to enhance NK cell infiltration in PDAC. A combination of IDO inhibitors and vaccine therapy is under investigation in PDAC, showing promise in overcoming immune resistance. However, IDO inhibition alone has shown limited effectiveness in PDAC [72]. Interestingly, Salmonella-based therapy coupled with the enzymatic depletion of tumor hyaluronan using PEGPH20 for targeting IDO has resulted in the complete regression of PDAC [187].

Another preclinical study explored the use of Salmonella typhimurium (ST) to overcome immune suppression in solid tumors. In a murine melanoma model, ST targeting the immune-suppressive factor STAT3 enhanced the immune response, reduced tumor growth, and improved survival when combined with tumor antigen vaccination. The approach was applied to PDAC, where physical barriers like fibrosis hinder therapeutic delivery. By combining ST with PEGPH20, which depletes hyaluronic acid in the TME, ST penetration and immune cell recruitment were improved, enhancing anti-tumor responses. This strategy showed promise for PDAC and other cancers with minimal toxicity [188].

Concurrently, therapy involving CCK-receptor antagonists has the potential to diminish tumor-associated fibrosis in PDAC [28], thereby boosting the effectiveness of immune checkpoint antibody therapy in PDAC mouse models [132].

Promising results from preclinical studies of ProAgio, an anti-alpha-v-beta-3 integrin cytotoxin, have led to its investigation as a potential therapeutic agent for reducing tumor-associated fibrosis in patients with PDAC. This research is currently being evaluated in a clinical trial registered under [189,190].

As an extension of these approaches, tumor fibrosis is an important prognostic factor in PDAC, and reducing fibrosis while boosting NK cell migration could enhance immune cell infiltration into tumors. The phenotyping of cancer-associated fibroblasts (CAFs), including inflammatory CAFs (iCAFs), myofibroblast-like CAFs (myCAFs), and mesenchymal CAFs (meCAFs), is crucial for understanding and targeting the TME. Interestingly, inhibitors of the JAK/STAT pathway have been shown to increase the myCAF-to-iCAF ratio, resulting in better tumor control [58]. Moreover, the presence of meCAFs has been linked to improved NK cell infiltration and responses to gemcitabine/nano-albumin-bound paclitaxel/anti-PD-1 therapy in PDAC patients [5]. A recent study by Francescone et al. showed that targeting by Netrin G1 protein (NetG1)-expressing CAFs in PDAC models reduced NK cell suppression and increased NK cell infiltration by boosting IL-15 and reducing IL-6. The number of myCAFs (αSMA + cells) did not change, suggesting that NK cells may interact more with IL-6-producing iCAFs, which suppress NK cell infiltration [191].

Furthermore, off-label therapeutic strategies combined with chemotherapy have been shown to enhance NK cell infiltration into tumors. For instance, Losartan, a conventional antihypertensive agent, was shown to reduce fibrosis by preventing the expression of TGF-*β* [192], and its efficacy was tested in combination with FOLFIRINOX in PDAC patients, yielding satisfying results [193].

#### 3.3.6. NK Cell Augmentation and Locoregional Treatments

Combining various locoregional treatments with NK cell augmentation may also enhance NK cell tumor infiltration in PDAC, while simultaneously increasing cytotoxicity. Modalities such as local hyperthermia using radiofrequency ablation, irreversible electroporation, and focused ultrasound-derived cavitation are being explored for their ability to improve NK cell infiltration [202].

Ionizing radiation can either boost anti-tumor immunity by creating a pro-inflammatory environment or suppress immune cells, promoting tumor growth and resistance. The effects depend on the delivery method and tumor type. Baude et al. explored studies addressing combining radiotherapy and NK cell-based therapies and suggested that this combination approach may lead to more effective tumor targeting and better therapeutic outcomes in solid tumors [214]. Several clinical trials are exploring the combination of allogeneic NK cell therapy and radiotherapy in patients with solid tumors [215]. Rahman et al. investigated this approach in a preclinical PDAC model, demonstrating that T-cell receptor alpha-beta (TCRαβ)-depleted NK/macrophage-enriched cell therapy, stimulated with IL-2 and zoledronic acid, significantly enhances PDAC cell-killing when combined with tumor-specific monoclonal antibodies and radiation therapy [194].

A preclinical study tested an in situ vaccination method combining radiotherapy, tumor-specific antibodies, and interleukin-2 (IL-2) in a mouse model. This approach, which has been trialed in metastatic melanoma patients, showed improved antitumor immunity, and could be applied to various metastatic cancers. The study also found that NK cells reduce regulatory T cells (Tregs) in non-targeted tumors, and depleting NK cells weakened the antitumor response, underscoring their key role in the treatment’s effectiveness [195].

A multi-center clinical trial evaluated a novel treatment strategy for heavily pre-treated, advanced metastatic PDAC patients. The approach combined low-dose chemo-radiation (nab-paclitaxel, gemcitabine, cyclophosphamide, and SBRT) to induce immunogenic cell death, followed by immunotherapies targeting both innate and adaptive immunity (aldoxorubicin, N-803, and PD-L1 t-haNK cells). This outpatient regimen showed promising results in survival and disease control, with prophylactic G-CSF or EPO excluded [196].

#### 3.3.7. Targeting Calcium Signaling and Diet in NK Cell Infiltration

Targeting calcium signaling is another promising approach to enhance NK cell infiltration. Identifying and targeting specific components of the Ca^2+^ toolkit through pharmacological agents or gene therapies may offer potential avenues for therapeutic intervention. In the context of PDAC treatment, both gemcitabine and 5-FU have been found to upregulate ORAi1, a key protein involved in SOCE, which influences tumor–TME interactions and enhances NK cell infiltration. These findings suggest a potential link between SOCE-mediated Ca^2+^ signaling and the response to chemotherapy in PDAC [73,136].

Furthermore, understanding the complex interaction between dietary factors, CCK signaling, and other elements such as hypoxia signaling can aid in the development of new therapeutic modalities to overcome resistance in managing PDAC [28,39].

#### 3.3.8. Targeting MicroRNAs for NK Cell Infiltration

Manipulating microRNAs offers a promising strategy for enhancing NK cell function. For instance, microRNA-301a, an onco-miRNA, is upregulated in human PDAC tissues [216], promoting NF-*κ*B activation and tumor growth. Inhibiting microRNA-301a or increasing Nkrf levels can reduce NF-*κ*B target gene expression and indirectly enhance NK cell infiltration, potentially slowing xenograft tumor growth [197]. MicroRNA-26a has been shown to inhibit the EMT process by downregulating EZH2 expression, inducing NK cell tumor infiltration, and improving the outcomes of immunotherapy in solid tumors [48,198]. Furthermore, **miR-26a** may offer a therapeutic approach for **PDAC** and other cancers by targeting the **E2F7/VEGFA axis**, which regulates angiogenesis and tumor growth. Restoring or mimicking miR-26a could help inhibit tumor progression, making it a promising candidate for future cancer therapies [199]. This strategy not only addresses the tumor’s invasive characteristics but also aims to enhance the efficacy of existing therapies by promoting a more favorable immune environment. Consequently, the exploration of microRNA-26a as a therapeutic agent in PDAC could represent a promising avenue for overcoming resistance and improving patient outcomes.

This table outlines the diverse modalities for activating NK cells in PDAC, focusing on three primary mechanisms: enhancing cytotoxicity, promoting NK cell proliferation, and increasing tumor infiltration. The first column lists the specific modalities employed, while the second column highlights the agents or strategies used as NK cell activators. The third column provides details on the type of study conducted (e.g., preclinical, clinical, or in vitro) and corresponding references. The fourth column summarizes the observed results, including improved NK cell function, increased anti-tumor activity, or enhanced infiltration into the tumor microenvironment. Although these modalities are not yet standard clinical approaches, they demonstrate potential for advancing NK cell-based therapies in PDAC treatment. For abbreviation definitions, please see the legend of Table 1.

## 4. Discussion

PDAC remains one of the most challenging malignancies due to its intrinsic phenotypic heterogeneity, complex TME, and associated therapeutic resistance. Despite certain genomic uniformity, PDAC tumors exhibit significant variability in immune landscape, cellular components, and therapeutic responses, which complicates treatment approaches. Standard therapies, such as chemotherapy and radiation, are insufficient on their own because they can induce treatment resistance and contribute to NK cell dysfunction over time. This highlights the urgent need for alternative strategies that not only overcome therapeutic resistance but also restore NK cell functionality, which is critical for building an effective immune response against PDAC.

Monitoring biomarker status before and during treatment for PDAC is crucial to understanding NK cell activity and optimizing therapy. However, repeated biopsies to assess NK cell status during treatment are not practical. Therefore, identifying surrogate, non-invasive diagnostic biomarkers is essential. A “Triple NK Cell Biomarker Approach”, which focuses on tumor features, TME factors, and NK cell suppression mechanisms, offers a promising pathway for the dynamic assessment of NK cell dysfunction. This approach can provide critical insights into the evolving immune landscape of PDAC and help guide the development of personalized therapies to enhance NK cell function and counteract treatment resistance.

Biomarkers like genomic alterations, such as KRAS, p53, and MYC mutations, as well as epigenetic changes like NAMPT overexpression and GATA6 downregulation, contribute to NK cell dysfunction and PDAC progression [22,52,88]. The TME, further exacerbates these challenges through factors such as TGF-β and immune checkpoints (e.g., PD-1, TIM-3, TIGIT), which impair NK cell function [71,73,77]. Promising strategies to counteract these barriers include STAT3 knockout, FAK inhibition, and targeting ECM components like CAFs and collagen using MMP inhibitors or CAF-targeting agents to enhance NK cell infiltration [55,58,170,212].

Combination therapies like immune checkpoint inhibitors with chemotherapy or locoregional therapeutic modalities like radiotherapy, also show potential to improve NK cell responses. Repurposing off-label drugs, such as metformin, losartan, diclofenac, and vitamin D analogs, offers another avenue for restoring NK cell function in PDAC [32,33,148,150,193].

Innovative strategies, such as NK cell engagers, CAR NK cells, and NK cell-based aptamer and vaccines, aim to enhance NK cell activity [70,78,157,167] but face challenges, including impaired cytotoxicity, reduced proliferation, poor tumor infiltration, and NK cell exhaustion due to the immunosuppressive TME. Solutions for CAR NK therapy includes engineering NK cells for better survival, using cytokines like IL-15, combining them with immune checkpoint inhibitors, and improving tumor infiltration through tumor-homing receptors or nanoparticles. BiKEs and TriKEs also show promise but face similar barriers, which can be addressed through structural modifications, cytokine support, and combining with immune modulators. NK cell-based vaccines can benefit from strategies used in CAR NK therapy, such as enhancing NK cell persistence and targeting specific tumor antigens more effectively. Additionally, microbiome-modulating therapies, such as using Salmonella-based approaches [181,187] or targeting dysbiosis, are gaining attention, although further research is needed to overcome challenges related to safety and clinical consistency.

## 5. Future Direction

A promising future direction involves using the “Triple NK Cell Biomarker Approach” in biomarker studies and clinical trial design. This approach combines tumor features, TME factors, and NK cell suppression mechanisms, offering a personalized strategy to enhance NK cell function and counteract resistance. Investigating off-label drugs and immune modulators could further improve NK cell activity. Additionally, utilizing aptamers to activate NK cells and targeting microbiota profiles as potential biomarkers in PDAC present exciting research avenues. By integrating these strategies with conventional treatments, NK cell-targeted therapies could significantly improve PDAC treatment outcomes.

## 6. Conclusions

The future of PDAC treatment lies in improving NK cell-based therapies, including NK cell engagers, CAR NK cells and NK cell vaccines, to overcome current limitations. By targeting the TME, enhancing NK cell survival, and optimizing vaccine strategies, we can improve the ability of NK cells to fight PDAC. Combining NK cell therapies with other strategies that address the tumor, TME, such as ECM modulation and NK cell suppression mechanisms, holds great potential for improving clinical outcomes in PDAC.

## Figures and Tables

**Table 2:** NK cell-related therapeutic modalities.

NK Cell Activators	Combinatory Modalities	Study Type [Reference]	Result
Epigenetic Modulators	EZH2 inhibitors	Preclinical [48]	Increased NK cell cytotoxicity
	HAT inhibitors	Review [54]	Increased NK cell cytotoxicity
	HDACs blockers	Preclinical, Review [53,54]	Increased NK cell cytotoxicity
	GSK3B combined to gemcitabine	Preclinical [53]	Increased NK cell cytotoxicity: Enhanced gemcitabine induced apoptosis
Metabolic Modulators	NAD+ supplementation+ STING agonists combined to NAMPT. Inhibitors or combined to metformin	Preclinical [69,151]	Increased NK cell cytotoxicity; Enhanced NAMPT and metformin sensitivity
	MYC targeting combined to Metformin	Preclinical [148]	Increased NK cell cytotoxicity; Sensitize CSCs to metformin
Cytokine Modulators	IL-10 blockers PEGylated human IL-10 (AM0010)	Clinical Trial [62,152]	Increased NK cell cytotoxicity
	IL-12 MCBD-IL12	Preclinical [153,154]	Increased NK cell cytotoxicity
	IL-21 +Cetuximab	Preclinical [47]	Increased NK cell cytotoxicity; regardless of KRAS mutation status
	CSF1/1R inhibitors	Preclinical [35,155,156]	Increased NK cell numbers and cytotoxicity
	DMXAA (STING agonist)	Preclinical [80,81]	Increased NK cell numbers and cytotoxicity
	NK cell combined to TGF-β neutralizers.	Preclinical [58,145]	Increased NK cell cytotoxicity
	vvDD-IL2 and vvDD-IL15 (cytokine-armed vaccinia viruses)	Preclinical [157]	Increased NK cell cytotoxicity
Targeted Combination Therapies	Neoantigen vaccine combined to TIGIT inhibitors	Preclinical [82]	Increased NK cell cytotoxicity
	Radiotherapy + FAK inhibitors +checkpoint inhibitors	Preclinical [158,159]	Increased NK cell cytotoxicity
	Durvalumab (PD-L1 inhibitor) + sotigalimab (CD40) agonist in combination with gemcitabine/nano-albumin-bound paclitaxel	Clinical trial [160]	Increased NK cell cytotoxicity
	Lenvatinib (VEFGR inhibitor) + Pembrolizumab (PD-1 inhibitor)	Clinical trial [76]	Increased NK cell cytotoxicity (decreased NK cell exhaustion)
	Lenvatinib (VEFGR inhibitor) + Pembrolizumab (PD-1 inhibitor)	Case report [161]	Complete response in PDAC patient after extensive prior treatment.
	PKM2 inhibitor + PD-L1 inhibitors	Preclinical [162]	Increased NK cell cytotoxicity
	GVAX + CSF-1R inhibitors	Preclinical [36]	Increased NK cell cytotoxicity
	IL-6 + PD-1 inhibitors	Clinical [61]	Increased NK cell cytotoxicity
Other Molecular Strategies	DGK-inhibitors	Preclinical [44]	Increased NK cell cytotoxicity
	Cbl knockdown	Preclinical [163]	Increased NK cell cytotoxicity
	CD73 inhibitors (antibodies or diclofenac)	Preclinical [33]	Increased NK cell cytotoxicity
	MIC inhibitors (anti MIC shedding)	Preclinical [65]	Increased NK cell cytotoxicity
	Targeting alternative splicing of CD155	Clinical [31]	Increased NK cell cytotoxicity
	Aptamer based therapies	Preclinical and clinical [164,165,166,167,168]	Increased NK cell cytotoxicity
Targeting NK cell Expansion	Anti CD3 or anti CD 52	Preclinical [169]	Enhanced NK cell proliferation
	STAT3 knock out	Preclinical [170]	Enhanced NK cell proliferation
NK cell Engageres	BIKEs or TriKEs	Preclinical [70,171]	Enhanced NK cell proliferation and cytotoxicity
	cam1615TEM8 (TriKE)	Preclinical [172]	Enhanced IL-15 induced NK cell proliferation and cytotoxicity
	IL-15/B7-H3 TriKEs-based immunotherapy	Preclinical [173]	Enhanced IL-15 induced NK cell proliferation and cytotoxicity
Adoptive NK cell therapies	EAAL + gemcitabine/Oxaliplatin + nimotuzumab	Case report [174]	Enhanced NK cell proliferation and cytotoxicity resulting in tumor regression
	NKG2D and NKp30 activating receptors drive allogenic NK cell therapy	Preclinical [175]	Enhanced NK cell proliferation, cytotoxicity, and tumor infiltration resulting in tumor regression
	Allogenic NK cell therapy + IRE	Clinical Trial [176]	Enhanced NK cell proliferation and tumor infiltration resulting in tumor regression
	Autologous NK cell gemcitabine/erlotinib	Preclinical [177]	Enhanced NK cell proliferation and tumor infiltration resulting in tumor regression
Targeting Tumor-Intrinsic Factors	EZH2 knockdown	Preclinical [46]	Enhanced NK cell tumor infiltration
	EZH2 inhibitors+ trametinib/palbociclib (senescence-inducing therapies)	Preclinical [46]	Enhanced NK cell tumor infiltration
NK cell Engineering and Tumor Targeting	NOX-A12 (CXC12 antagonist) combined to pembrolizuma	Review and Clinical Trial [38,125]	Enhanced NK cell tumor infiltration and cytotoxicity
	NRP-bodies	Preclinical [39]	Enhanced NK cell tumor infiltration
	ROBO1) CAR NK-92 cells + brachytherapy	Preclinical [78]	Enhanced NK cell tumor infiltration and tumor regression
	PSCA-targeted CAR NK cells	Preclinical [178]	Enhanced NK cell tumor infiltration and tumor regression
	PSCA CAR-S15 NK cells	Clinical [179]	Safe and effective (tumor regression)
	cGMAP (STING agonist) combined to NK CAR cells	Preclinical [180]	Enhanced NK cell tumor infiltration
	ROBO1-specific CAR NK cells	Clinical [59]	Safe and effective (stable disease)
ECM Remodeling	MMP-9 inhibitor	Preclinical [55]	Enhanced NK cell cytotoxicity
	Combined Gli2/Gli3 deletion in fibroblasts	Preclinical [96]	Enhanced NK cell tumor infiltration and tumor regression
	bHs-ST (bacterial hyaluronidase produced by Salmonella typhimurium)	Preclinical [181]	Enhanced NK cell tumor infiltration
	PEGPH20 (hyaluronidase) combined to radiotherapy	Preclinical [182]	Enhanced NK cell tumor infiltration and increased radiosensitivity in high hyaluronan tumor
	IPI-269609 (hedgehog signaling inhibitor)	Preclinical [183]	Enhanced NK cell tumor infiltration and tumor regression preclinically
	IPI-926 (hedgehog inhibitor, SMO antagonist)	Preclinical [184]	Normalized tumor vascularization, enhanced drug delivery and NK cell infiltration
	FAK inhibitors	Preclinical [49,50,158,159,185]	Enhanced NK cell tumor infiltration, chemo and radiosensitization
	Vitamin D analogs	Preclinical and clinical [30,186]	Enhanced NK cell infiltration, gemcitabine efficacy
	ATRA (All-Trans Retinoic Acid)	Preclinical [77]	Targeting PSCs and rediced ECM stiffness, teoritically enhanced NK cell infiltration
Innovative and Off-Label Therapeutic Strategies to Promote NK Cell Infiltration	BRD4 targeting (bromodomain inhibitor)	Preclinical [56]	Enhanced NK cell infiltration in metastatic prone areas like the liver
	shIDO-ST (*Salmonella-*based therapy targeting the immunosuppressive molecule indoleamine 2,3-dioxygenase (IDO) + PEGPH20	Preclinical [187]	Enhanced NK cell infiltration and complete regression of PDAC
	*Salmonella-*based vaccine (STAT3 targeting)	Preclinical [188]	Enhanced NK cell infiltration and cytotoxicity, Tumor regression in mice
	CCK-receptor antagonist + immune checkpoint inhibitors	Preclinical [132]	Enhanced NK cell infiltration and immunotherapy sensitivity
	ProAgio (anti-alpha-v-beta-3 integrin cytotoxin)	Preclinical and Clinical Trial (Phase 1) [189,190]	Enhanced NK cell infiltration and tumor regression
	JAK/STAT inhibitors	Preclinical [5,58]	Enhanced NK cell infiltration and tumor regression, chemotherapy sensitivity
	NetG1 blocking (using neutralizing antibody)	Preclinical [191]	Enhanced NK cell cytotoxicity and infiltration and tumor regression
	Losartan combined to FOLFIRINOX	Preclinical and clinical [192,193]	Enhanced NK cell infiltration and tumor regression, induced chemotherapy sensitivity
NK Cell Augmentation and Locoregional Treatments	Allogenic NK cell therapy + tumor specific monoclonal antibody + Radiotherapy	Preclinical [194]	Enhanced NK cell infiltration and tumor regression
	In-situ vaccination + tumor specific monoclonal antibody + IL-2 + Radiotherapy	Preclinical [195]	Enhanced NK cell infiltration and tumor regression
	Aldoxorubin + N-803 (IL-15 super agonist) + PDL1t-haNK (PD-L1 NK cell therapy) following chemo-radiotherapy	Clinical [196]	Enhanced NK cell infiltration. Safe and effective (disease stability)
Targeting MicroRNAs for NK Cell Infiltration	MicroRNA-301a inhibition or Nkrf upregulation	Preclinical [197]	Reduce NF-*κ*B target gene expression and indirectly enhance NK cell infiltration, slowed xenograft tumor growth
	MicroRNA-26a mimics	Preclinical [198,199]	Enhanced NK cell infiltration by inhibiting EMT, increased the immunotherapy sensitivity

## Data Availability

The data presented in this study are available on request from the corresponding author.
